# Pulmonary function changes after sublobar resection in patients with peripheral non-subpleural nodules

**DOI:** 10.1186/s12893-022-01828-0

**Published:** 2022-11-11

**Authors:** Kun-Peng Feng, Zi-Qing Shen, Chun Xu, Cheng Ding, Yu Feng, Xin-Yu Zhu, Bin Pan, Xin-Yu Jia, Jun Zhao, Chang Li

**Affiliations:** 1 Department of Thoracic Surgery, The First Affiliated Hospital of Soochow University, Medical College of Soochow University, Suzhou, 215000 China; 2 Institute of Thoracic Surgery, The First Affiliated Hospital of Soochow University, Suzhou, China

**Keywords:** Peripheral lung cancer, Pulmonary function, Sublobar resection, Video-assisted thoracoscopic surgery

## Abstract

**Background:**

In the treatment of peripheral early-staged lung cancer and benign lesions, segmentectomy and wedge resection are both reliable treatment methods. It is debatable that how much pulmonary function will be lost after different sublobar resection in the treatment of early-staged deep-located peripheral NSCLC (non-small cell lung cancer). The purpose of this study was to explore postoperative pulmonary function changes of sublobar resection in enrolled patients with non-subpleural peripheral nodules.

**Methods:**

We collected clinical data of patients undergoing VATS (video-assisted thoracoscopic surgery) segmentectomy or wedge resection for single nodule. These nodules were confirmed as peripheral non-subpleural nodules by preoperative 3D imaging. Patients were divided into two groups according to the operation procedure. Demographic characteristics, pulmonary function, postoperative outcomes, and others were collected. All data was gathered at the First Affiliated Hospital of Soochow University. Outcomes after wedge resection were compared with those after segmentectomy resection.

**Results:**

A total of 88 patients were included in this study, including 46 patients with VATS wedge resection and 42 patients with VATS segmentectomy. No difference was detected when comparing FEV_1_ (forced expiratory volume in 1 s) loss between these two groups (17.6 ± 2.1%, wedge resection vs. 19.4 ± 5.4%, segmentectomy, P = 0.176). FVC (forced vital capacity) loss (8.7 ± 2.3%, wedge resection vs. 17.1 ± 2.2%, segmentectomy, P < 0.001) and MVV (maximum ventilatory volume) loss (11.5 ± 3.1%, wedge resection vs. 20.6 ± 7.8%, segmentectomy, P < 0.001) in segmentectomy group was significantly higher than those in wedge resection group. Discrepancies were investigated when comparing duration of surgery (70 ± 22 min, wedge resection vs. 111 ± 52 min, segmentectomy, P = 0.0002), postoperative drainage (85 ± 45 mL, wedge resection vs. 287 ± 672 mL, segmentectomy, P = 0.0123), and treatment hospitalization expenses [35148 ± 889CNY, wedge resection vs. 52,502 (38,276–57,772) CNY, segmentectomy, P < 0.0002]. No significant difference was found between air leak time (1.7 ± 0.7 days, wedge resection vs. 2.5 ± 1.7 days, segmentectomy, P = 0.062) and hospitalization time (2.7 ± 0.7 days, wedge resection vs. 3.5 ± 1.7 days, segmentectomy, P = 0.051).

**Conclusions:**

For patients with peripheral non-subpleural nodules, we observed that patients who underwent wedge resection had less lung function loss than those who underwent segmentectomy when their lung function was reviewed at the 6th month after surgery. Patients undergoing wedge resection had partial advantages over patients with segmental resection in terms of hospitalization cost, operation time and postoperative drainage, etc. Wedge resection, as a treatment for peripheral non-subpleural pulmonary nodules, seemed to have more advantages in preserving patients’ pulmonary function.

## Introduction

In previous cognition, sublobar resection was relegated as a compromised treatment, only for those patients who had poor pulmonary function or with comorbidities [1, 2]. Researchers pointed out that in the treatment of peripheral early-staged lung cancer and benign lesions, segmentectomy is a reliable treatment method, and OS (overall survival) is not inferior to traditional lobectomy [3–5]. The NCCN guidelines shared the same view that sublobar resection is a reliable treatment for early lung cancer [6]. Furthermore, scholars conducted a randomized controlled trial to confirm that segmentectomy is not inferior to lobectomy in terms of prognosis (Trial No. JCOG0802/WJOG4607L). The current research progress confirms that in the patients who underwent segmentectomy and lobectomy, there is no significant difference in incidence of complications between these two groups [7].

**Fig. 1:**
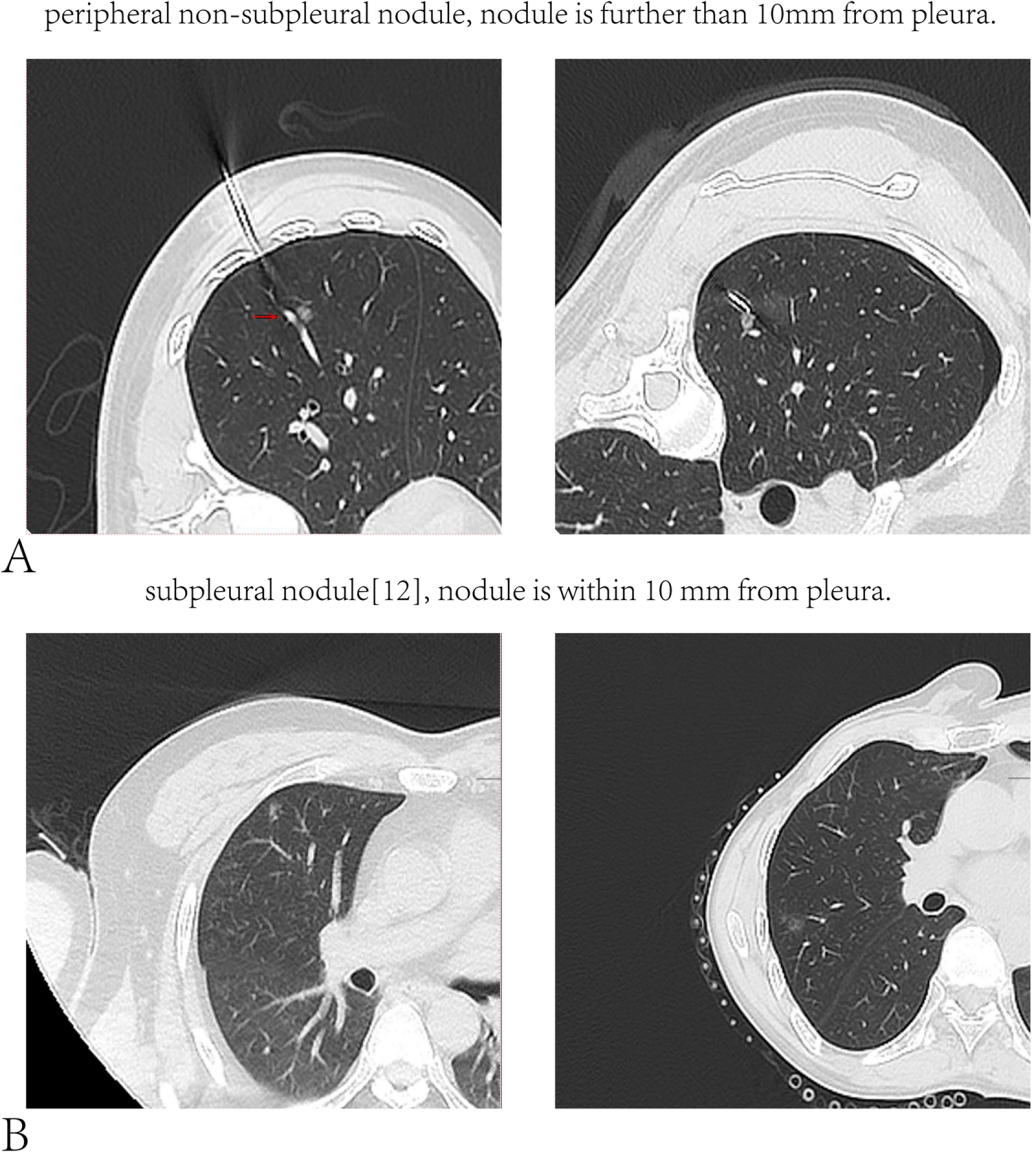
Peripheral pulmonary nodules can be divided into two types according to the distance between the nodules and the pleura [12], that is, subpleural nodules (nodule is within 10 mm from pleura) and non-subpleural nodules (nodule is further than 10 mm from pleura)

When different types of sublobar resection can be used to treat early staged superficial NSCLC, and both can achieve similar OS or recurrence-free survival [8, 9]. It has been documented that VATS wedge resection can best preserve pulmonary function with slight spirometry change as well as VATS mediastinal surgery [10]. Similar views also pointed out that VC (Vital capacity) of wedge resection can recover to the preoperative VC level at 12th month after procedure [11]. However, most of cases undergoing VATS segmentectomy included in previous studies were confirmed as patients with subpleural nodules (Fig. 1) [12]. It is debatable that how much pulmonary function will be lost after extended wedge resection in the treatment of early-staged deep-located peripheral NSCLC.

With the progress of imaging technology, especially the application of CT, more and more early-staged lung cancer has been detected. Some early-staged NSCLC appears as non-subpleural peripheral pulmonary nodules (Fig. 1). Thoracic surgeons usually adopt extended wedge resection or segmentectomy to remove these nodules. However, segmentectomy is technically more demanding than wedge resection because the surgeon will have to carefully identify segmental pulmonary vessels and bronchi [13, 14]. Moreover, segmentectomy has more concerns about the extension of operation time and postoperative air leak time, and so on [15, 16]. Various reasons mentioned above have limited the promotion of segmentectomy, and the impact of segmentectomy on postoperative pulmonary function is controversial.

The purpose of this study was to observe postoperative changes of pulmonary function, and to compare postoperative outcomes (thoracic drainage, treatment cost and air leak time, etc.) in patients with peripheral non-subpleural nodule undergoing VATS wedge resection and VATS segmentectomy.

## Methods and patients

**Fig. 2:**
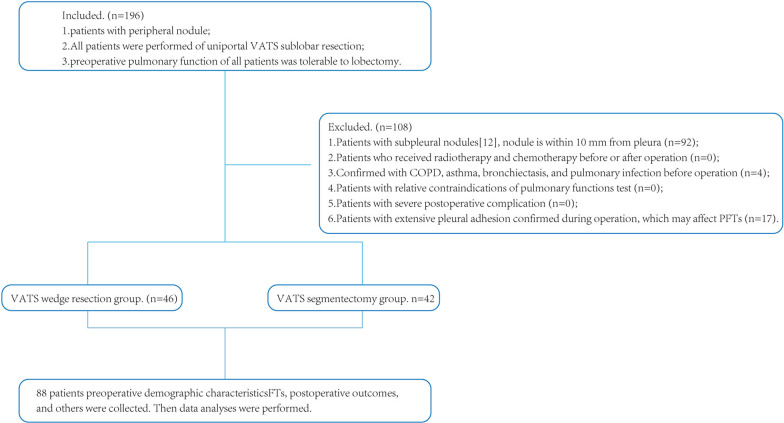
Study flow chart

Patients who underwent uniportal VATS sublobar resection at The First Affiliated Hospital of Soochow University between January 2021 and May 2021 were retrospectively included in this study, a total of 192 patients were included according to the inclusion criteria, and 104 patients were excluded according to the exclusion criteria, final 88 patients were included in the study (Fig. 2). The study was approved by the Institutional Review Board of the hospital (Ethical approval no. 2022077). 88 patients were divided into two groups according to the surgical procedures, VATS wedge resection group, and VATS segmentectomy group. Study data included: patient age, sex, smoking history, pathological types, distance from nodule to pleura, length of stapler on CT scan (3 months after surgery), preoperative and postoperative PFTs (pulmonary function tests), operation characteristics (operating time, intraoperative blood loss), postoperative recovery (chest drainage, postoperative complications, air leak time, hospital stay), treatment and hospitalization expenses.

### Inclusion criteria

(1) Patients with peripheral nodules; (2) Regardless of the number of nodules, a patient was performed only a single type of surgery; (3) All patients were performed of VATS sublobar resection (wedge resection, segmentectomy); (4) All operations were under uniportal VATS; (5) The preoperative pulmonary function of all included patients was tolerable to lobectomy.

### Exclusion criteria

(1) Patients with subpleural nodules (nodule is not further than 10 mm from the pleura). (2) Those patients who received radiotherapy and chemotherapy before operation. (3) The patients confirmed with COPD (Chronic Obstructive Pulmonary Disease), asthma, bronchiectasis, and pulmonary infection before operation. (4) Patients with relative contraindications of pulmonary function test, like acute myocardial infarction within 1 week, or history of neurosurgery within 4 weeks. (5) Patients with severe postoperative complications; (6) Patients with extensive pleural adhesion confirmed during operation, which may affect the preoperative pulmonary function. (6) Patients received radiotherapy and chemotherapy after operation.

### Surgical technique

#### Patient position

Lateral position, healthy side down.

#### Anesthesia

General anesthesia, double-cavity endotracheal intubation, single-lung ventilation.

#### Patient position

The patient was set in lateral position, healthy side down.

#### Surgical procedure

All patients are divided into two groups according to the surgical procedure. All patients were performed a single incision whose size was 3–6 cm in the fourth or fifth intercostal space of the anterior axillary line. The surgical procedure was decided according to the preoperative imaging data, intraoperative rapid pathology, and operation difficulty, surgeon’s habits or so on.

#### Segmentectomy’s procedure

All patients underwent chest CT scan after admission. We used Exoview system to make preoperative 3D reconstruction. Then we used ICG (indocyanine green) fluorescence method to identify the IBL (intersegmental boundary line) and perform anatomical segmentectomy. Next, leakage test was performed. If significant air leakage is found, 4-0 Prolene suture was used for repair. Lymph nodes were grouped and resected or sampled according to the Chinese Guidelines for the Diagnosis and Treatment of Primary Lung Cancer [17]. A 28-F chest drainage tube was placed at the surgical port after surgery.

#### Wedge resection’s procedure

**Fig. 3:**
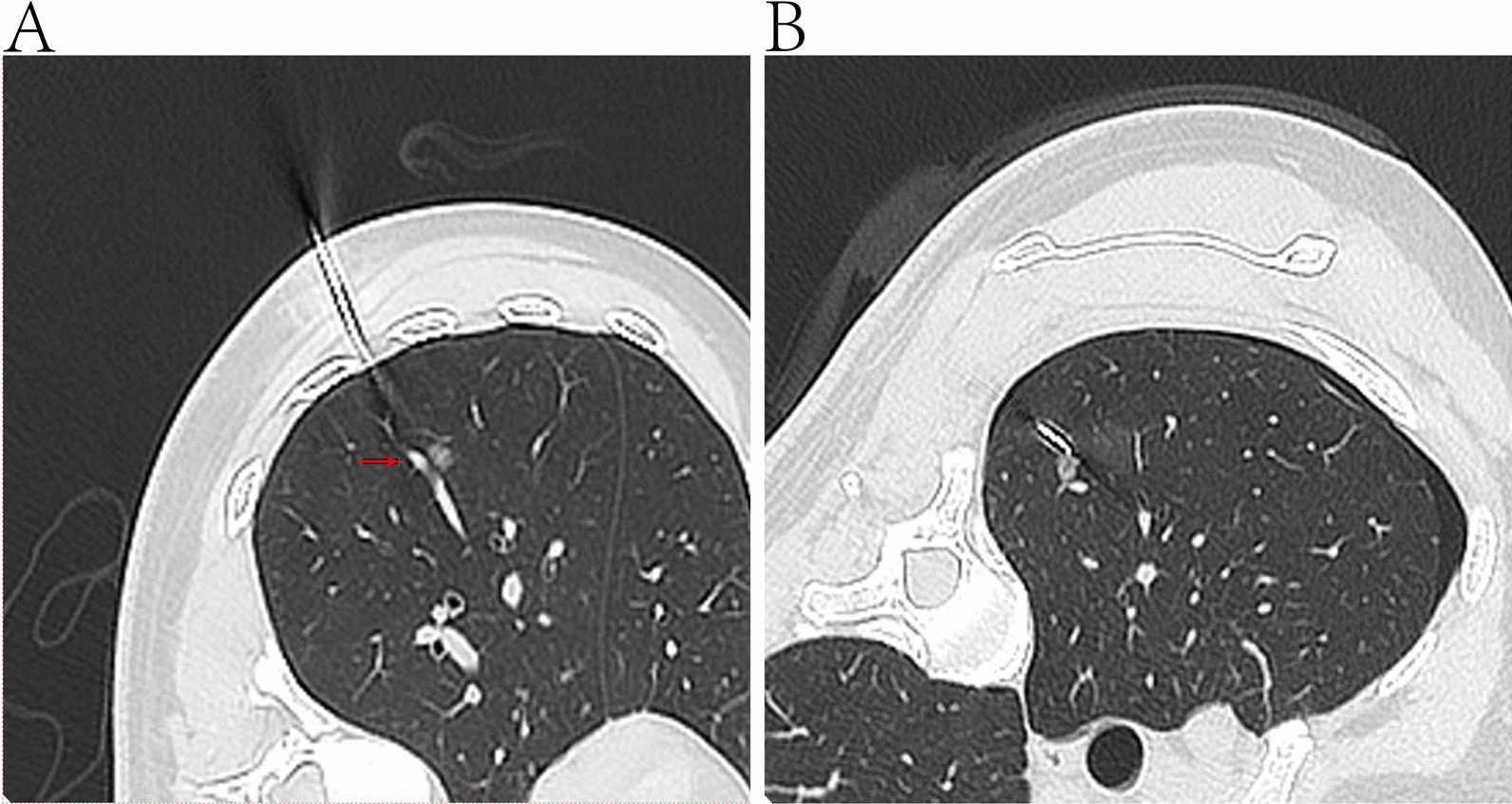
CT guided localization may be performed 30 min before the operation in order to achieve accurate resection according to the needs of the operation

Preoperative preparation was not significantly different from segmentectomy. According to the needs of the operation, CT guided localization may be performed 30 min before the operation in order to achieve accurate resection (Fig. 3). Wedge resection was performed using staplers. Margins were confirmed intraoperatively, most cases obtained a sufficient resection margin, and additional resection were added if margin was not enough. The patient was placed a chest tube postoperatively, then returned to the ward.

Every operation we performed the resection of tumor according to the principles of oncology, the length of the resection margins was guaranteed to be greater than the tumor diameter or greater than 2 cm, and all samples were sent for rapid frozen section to clarify the pathology and ensure negative margins. Patients included in this study all had complete tumor resection with negative margins.

*Postoperative management *All patients returned to the thoracic ward after surgery. Patients were guided to make breathing exercises, and encouraged to ambulate, in order to reduce the post-operative complications, thus discharge early and improve pulmonary function and QOL (quality of life). Patients whose chest tube drainage was less than 200 mL/day, radiograph confirmed no pneumothorax, and cough without air leak were allowed to remove their chest tube.

*Calculation method of lung function loss *All patients were performed pulmonary function tests before and 6 months after surgery. Pulmonary function tests include: FVC, FEV_1,_ MVV. The pulmonary function loss was calculated as follows (take FVC loss as an example): FVC loss = (preoperative FVC − postoperative FVC)/preoperative FVC × 100% [10].

*Data analyses* We used SPSS 25.0 software (Statistical Package for the Social Sciences, Chicago, IL, USA) for data analysis. All data results were expressed as mean value ± standard deviation. Multivariate analysis of variance (ANOVA) was used to assess whether categorical variables such as patient gender had effect on FVC loss and FEV_1_ loss. Independent sample t-test was applied for the measurement data conforming to normal distribution while Mann–Whitney U test was used for that not conforming to normal distribution. When *P* value of less than 0.05, statistical significance was accepted.

## Result

Altogether 88 patients were enrolled in the study, 46 patients undergone VATS wedge resection, 42 VATS segmentectomy. Patients’ characteristics are shown in Table 1. Wedge resection has a shorter duration of surgery (70 ± 22 min, wedge resection vs. 111 ± 52 min, segmentectomy, P = 0.0002), less postoperative drainage (85 ± 45 mL, wedge resection vs. 287 ± 672 mL, segmentectomy, P = 0.0123) and less hospitalization cost [35148 ± 889CNY, wedge resection vs. 52,502 (38,276–57,772) CNY, segmentectomy, P = 0.0002] than segmentectomy. No significant differences were detected between two groups in the air leak time (1.7 ± 0.7 days, wedge resection vs. 2.5 ± 1.7 days, segmentectomy, P = 0.062) and hospitalization time (2.7 ± 0.7 days, wedge resection vs. 3.5 ± 1.7 days, segmentectomy, P = 0.051).

**Table 1:** Clinical details of all patients

	Wedge resection	Segmentectomy	P value
n	46	42	
Age (year)	46.9 ± 13.0	46.5 ± 12.2	0.724
Gender			0.583
Female	30	24	
Male	16	18	
Preoperative imaging diagnosis			0.426
Pure GGN	30	32	
Mixed GGN	16	10	
Target lung segment			
S^1^		6	
S^1+2^		14	
S^4+5^		4	
S^6^		10	
S^9+10^		8	
Intraoperative frozen section diagnosis			
AHH	0	0	
AIS	34	28	
MIA	6	6	
IAC	0	0	
Benign lesion	6	8	
Tumor diameter	12.4 ± 3.6	10.2 ± 4.6	0.095
Distance from nodule to pleura (Mm)	24.0 ± 5.4	28.1 ± 11.8	0.347
Smoking history			
Yes	4	4	
No	42	38	
Duration of surgery (min)	70 ± 22	111 ± 52	0.0002***
Postoperative drainage (mL)	85 ± 45	287 ± 672	0.0123*
Air leak time (days)	1.7 ± 0.7	2.5 ± 1.7	0.062
Length of hospitalization (days)	2.7 ± 0.7	3.5 ± 1.7	0.051
Complication	0	0	
Pathological type			
AHH	0	2	
AIS	34	26	
MIA	6	6	
IAC	0	0	
Benign lesion	6	8	
Treatment and hospitalization expenses (CNY)	35,148 ± 889	52,502 (38,276–57,772)	0.0002***

Wedge resection has a shorter duration of surgery, less postoperative drainage and less hospitalization cost than segmentectomy. No significant differences were detected between two groups in the air leak time and hospitalization time. No significant difference was found in the distance from nodule to pleura between the two groups, indicating that the depth of pulmonary nodules was similar between the two groups. The length of stapler trace on chest CT 3 months after operation was measured for each patient, traces of staplers on chest CT scan were relatively more obvious when patients who underwent segmental pneumonectomy came for review 3 months after operation

*AHH* atypical adenomatous hyperplasia, *AIS* adenocarcinoma in situ, *MIA* minimally invasive adenocarcinoma, *IAC* invasive adenocarcinoma cancer

**Fig. 4:**
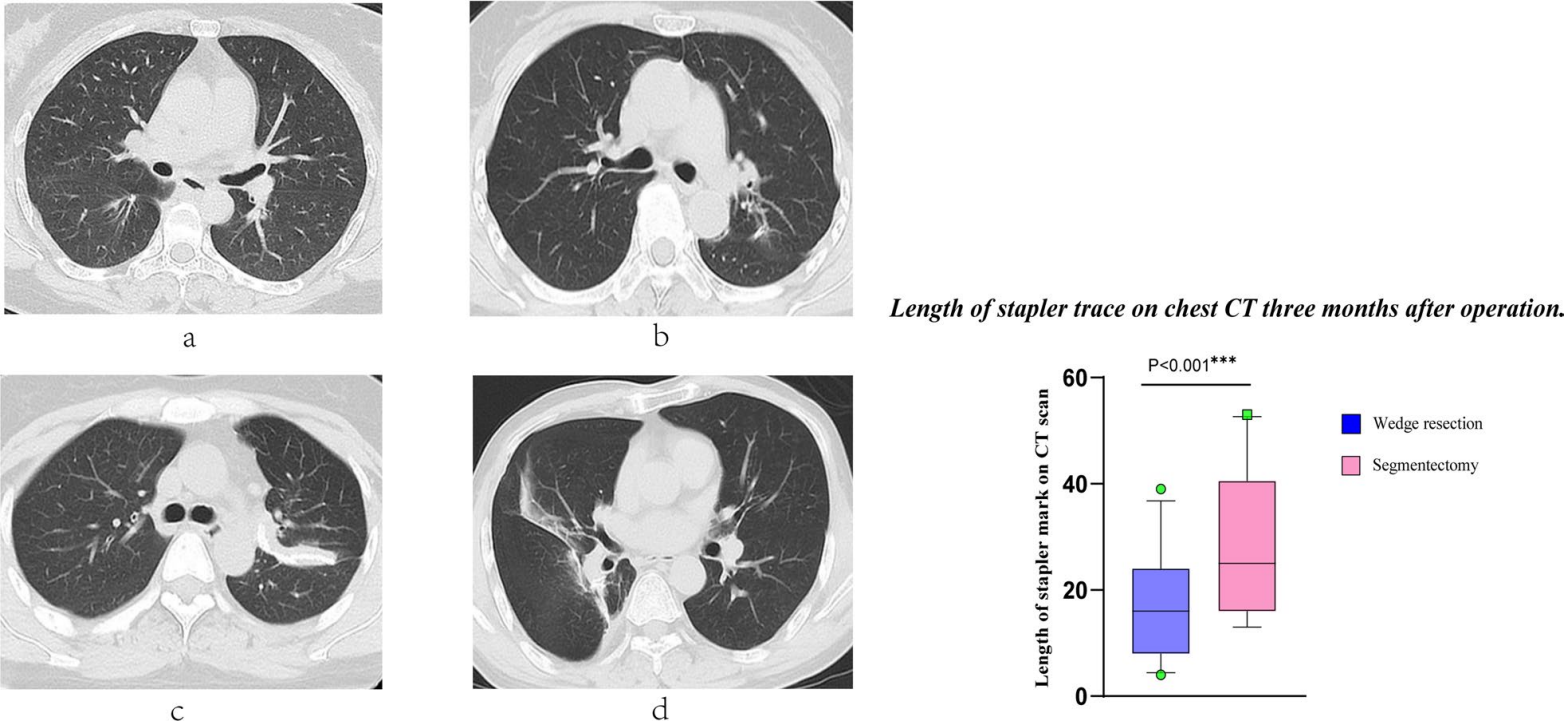
When patients undergoing wedge resection were reviewed at 3 months postoperatively, no significant stapler traces were detected on chest CT in most patients. Representative of these patients were shown in figures **a** and **b**, whose re-expansion of residual lung was satisfactory. Some patients represented by figure **c** and **d** had more pronounced and dense stapler traces visible on chest CT when reviewed 3 months after VATS segmentectomy, which might affect the re-expansion of residual lung

No significant difference was found in the distance from nodule to pleura between the two groups (24.0 ± 5.4 mm, wedge resection vs. 28.1 ± 11.8 mm, segmentectomy, P = 0.347), indicating that the depth of pulmonary nodules was similar between the two groups. The length of stapler trace on chest CT 3 months after operation was measured for each patient (Fig. 4), then the data collected from these two groups was compared, and evident difference was discovered (16.7 ± 9.5 mm, wedge resection vs. 27.7 ± 13.4 mm, segmentectomy, P < 0.001). The number of staplers used in segmental resection group was also significantly higher than that in wedge resection group (2.5 ± 0.7 staplers, wedge resection vs. 5.2 ± 1.6 staplers, segmentectomy, P < 0.001).

Preoperative pulmonary function and postoperative changes of pulmonary function are shown in Table 2. No discrepancy was noticed when FEV_1_ loss between two groups were compared (17.6 ± 2.1%, wedge resection vs. 19.4 ± 5.4%, segmentectomy, P = 0.176). FVC loss after segmentectomy was prominently greater than after wedge resection (8.7 ± 2.3%, wedge resection vs. 17.1 ± 2.2%, segmentectomy, P < 0.001), similar results are shown in MVV loss (11.5 ± 3.1%, wedge resection vs. 20.6 ± 7.8%, segmentectomy, P < 0.001).

**Table 2:** Preoperative pulmonary function and postoperative changes of pulmonary function after VATS resections

	Wedge resection	Segmentectomy	P value
Preoperative pulmonary function			
Pre FEV_1_ (L)	2.19 ± 0.54	2.80 ± 0.72	0.003**
Pre FVC (L)	2.50 ± 0.56	3.24 ± 0.77	< 0.001***
Pre FEV_1_/FVC (%)	87.1 ± 2.2	86.0 ± 1.0	0.185
Pre MVV (L/min)	106.4 ± 12.5	129.4 ± 17.4	< 0.001***
Postoperative pulmonary function			
Post FEV_1_ (L)	1.80 ± 0.44	2.26 ± 0.62	0.007**
Post FVC (L)	2.27 ± 0.50	2.68 ± 0.65	0.025*
Post FEV_1_/FVC (%)	78.6 ± 2.1	83.5 ± 4.7	< 0.001***
Post MVV (L/min)	95.2 ± 6.4	103.7 ± 11.7	0.003**
Pulmonary function loss			
FEV_1_ loss (%)	17.6 ± 2.1	19.4 ± 5.4	0.176
FVC loss (%)	8.7 ± 2.3	17.1 ± 2.2	< 0.001***
MVV loss (%)	11.5 ± 3.1	20.6 ± 7.8	< 0.001***

Postoperative FVC, FEV_1_ and MVV level at 6 months after operation decreased in both groups. No significant statistical differences were found on postoperative FEV_1_ loss after different types of sublobar resection, FVC and MVV loss after segmentectomy was significantly greater than after wedge resection

**Fig. 5:**
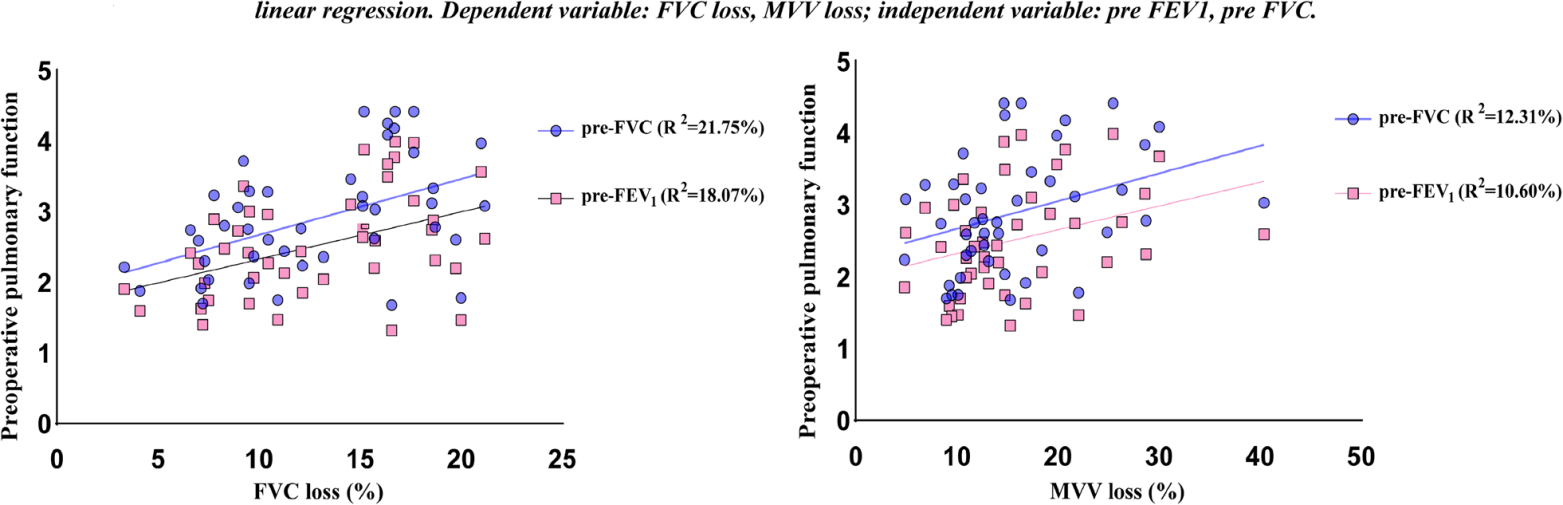
Linear regression result showed that the change of preoperative pulmonary function was not able to trigger the change of FVC loss and MVV loss

After analysis of these data, it was found that the preoperative pulmonary function was statistically different between the two groups of patients. To explore how much these two factors contributed to FVC loss and MVV loss, two factors were included in a linear regression with the dependent variable as FVC loss and MVV loss. Linear regression result showed that the change of preoperative pulmonary function was not able to trigger the change of FVC loss and MVV loss. Result was showed in Fig. 5.

Independent sample t-test showed that wedge resection could cause less FVC and MVV loss relative to segmentectomy (P < 0.001, P < 0.001). FVC loss after segmentectomy was significantly greater than after wedge resection.

## Discussion

Sublobar resection as a surgical procedure that can be accepted by thoracic surgeons for the treatment of early-staged NSCLC is becoming prevalent. Many literatures have reported that sublobar resection is suitable for small pulmonary nodules with GGO [18]. VATS is often preferred for patients with subpleural nodule, evidence has accumulated that the early and late outcomes of VATS are comparable or even superior to those of open thoracotomy [19]. However, it is still indeterminate which kind of sublobar resection has more or less effects on lung function after surgery. While segmentectomy requires a high level of thoracoscopic operation and familiarity with anatomy for thoracic surgeons. For various reasons, thoracic surgeons sometimes fall into a dilemma in preoperative evaluation.

Our results showed that the pulmonary function at 6 months after operation decreased in both groups. No significant statistical differences were found on postoperative FEV_1_ loss after different types of sublobar resection (Table 2), segmentectomy could cause more FVC and MVV loss to wedge resection. Previous study showed that FEV_1_ will begin to recover around 6 months after wedge resection, and VC then recovered to near the preoperative level after 12 months [11]. Therefore, we speculated that if the PFTs was performed 1 year after operation, pulmonary function of patients in wedge resection group and segmental resection group might return to a similar level. So, we chose to measure the postoperative pulmonary function 6 months after operation, this made it possible to observe which surgical procedure has a greater early effect on patients’ postoperative lung function. This follow-up scheme has also been adopted by other scholars [10].

No significant divergences were discovered when comparing FEV_1_ loss (19.4 ± 5.4% segmentectomy vs. 17.6 ± 2.1% wedge resection, P = 0.176), while all patients underwent uniportal VATS wedge resection or segmentectomy. Previous studies have confirmed that the FEV_1_ loss in patients undergoing thoracotomy is significantly greater than that in patients undergoing thoracoscopic surgery [20], we considered that the changes of FEV_1_ after pneumonectomy might be related to surgical approach, similar views have also been accepted by other scholars [21].

Significant difference in FVC loss was detected between wedge resection group and segmentectomy group (Table 2). We believed that the application of linear cutting stapler in the intersegmental plane will limit the re-expansion of the remaining lung segments, same view has also been clarified in other literature [22]. Although the cutting stapler was used to complete lung resection in both groups, re-expansion of residual lung might be more difficult in the segmentectomy group than in the wedge resection group. Several patients who underwent segmentectomy had more pronounced and dense stapler traces visible on chest CT when reviewed 3 months after surgery, which might restrict the re-expansion of remaining lung, data analysis also confirmed our notion (Fig. 4). Partial thoracic surgeons were accustomed to assuming that the amount of postoperative loss of lung function is proportional to the number of resected lung segments when performing preoperative assessments. However, previous studies have noticed that due to various reasons (different pulmonary function interval time, impacts caused by surgical incisions and different re-expansion after various extent of resection, etc.), postoperative pulmonary function might be inaccurately predicted by segment counting method [23–25]. It is difficult to determine precisely whether a patient is fit for VATS segmentectomy. Interestingly, a study has pointed out that not all segmentectomies preserve pulmonary function well, even sometimes lobectomy preserves more lung function relative to segmentectomy [26], this study results also equally corroborated our view. MVV loss showed similar results to FVC loss between the two groups (Table 2). MVV index is affected by airway lung tissue compliance, lung volume and respiratory muscle function at the same time [27]. Such a result might have a relationship with the surgical procedure. Segmentectomy involved processing of the intersegmental plane and was more complex to operate than wedge resection, then the use of staplers was generally more than wedge resection. The above-mentioned factors may cause FVC loss, it might also have a similar impact on the loss of MVV.

The trial No. JCOG0802 mentioned that segmentectomy could cause longer postoperative air leakage relative to lobectomy. In this study, we did not find a significant statistical difference in postoperative air leakage time between the two groups (Table 1). We believed that the reduction of air leak time after segmentectomy was partly related to technological advances (including the use of intraoperative adhesive materials as well as staplers, preoperative three-dimensional reconstruction, etc.) and the proficiency of the operating surgeon in performing thoracoscopic segmentectomy.

Compared with the segmentectomy group, the wedge resection group had less operation time, less thoracic drainage and less treatment and hospitalization expenses.

It has been documented that long-term respiratory exercise or exercise after discharge can improve lung function [28]. It will be considered in future studies.

## Conclusions

For patients with peripheral non-subpleural nodules, when comparing their lung function at 6th month after surgery, the loss of FEV_1_ in patients undergoing wedge resection was not prominently different to that of segmentectomy, wedge resection seemed to have more advantages in preserving patients’ FVC and MVV. Wedge resection also showed recognized ability in other aspects (saving treatment costs, reducing operation time, etc.). VATS wedge resection for peripheral non-subpleural nodules were not inferior to that of VATS segmentectomy. Whether wedge resection is superior to segmentectomy requires future comparison of the oncologic outcomes of the two surgical modalities in patients.

## Data Availability

The data supporting this study can be obtained from the corresponding author [Chang Li]; As the research data involve patient privacy and informed consent, the data will not be disclosed.

## Abbreviations

NSCLC: Non-small cell lung cancer
VATS: Video-assisted thoracoscopic surgery
FEV_1_: Forced expiratory volume in 1 s
FVC: Forced vital capacity
MVV: Maximum ventilatory volume
OS: Overall survival
VC: Vital capacity
PFTs: Pulmonary function tests
COPD: Chronic Obstructive Pulmonary Disease
ICG: Indocyanine green
IBL: Intersegmental boundary line
AHH: Atypical adenomatous hyperplasia
AIS: Adenocarcinoma in situ
MIA: Minimally invasive adenocarcinoma
IAC: Invasive adenocarcinoma cancer)

## References

[1] Ginsberg RJ, Rubinstein LV (1995). Randomized trial of lobectomy versus limited resection for T1 N0 non-small cell lung cancer. Lung Cancer Study Group. Ann Thorac Surg.

[2] Martin-Ucar AE, Delgado Roel M (2013). Indication for VATS sublobar resections in early lung cancer. J Thorac Dis.

[3] Harada H, Okada M, Sakamoto T, Matsuoka H, Tsubota N (2005). Functional advantage after radical segmentectomy versus lobectomy for lung cancer. Ann Thorac Surg.

[4] Kim HK, Han KN (2017). Uniportal video-assisted thoracoscopic surgery segmentectomy. Thorac Surg Clin.

[5] Lopez-Pastorini A, Koryllos A, Schnell J, Galetin T, Defosse J, Schieren M, Ludwig C, Stoelben E (2018). Perioperative outcome after open and thoracoscopic segmentectomy for the treatment of malignant and benign pulmonary lesions: a propensity-matched analysis. J Thorac Dis.

[6] Ettinger DS, Wood DE, Aggarwal C, Aisner DL, Akerley W, Bauman JR, Bharat A, Bruno DS, Chang JY, Chirieac LR (2019). NCCN guidelines insights: non-small cell lung cancer, Version 1.2020. J Natl Compr Cancer Netw.

[7] Suzuki K, Saji H, Aokage K, Watanabe SI, Okada M, Mizusawa J, Nakajima R, Tsuboi M, Nakamura S, Nakamura K (2019). Comparison of pulmonary segmentectomy and lobectomy: safety results of a randomized trial. J Thorac Cardiovasc Surg.

[8] Fiorelli A, Caronia FP, Daddi N, Loizzi D, Ampollini L, Ardo N, Ventura L, Carbognani P, Potenza R, Ardissone F (2016). Sublobar resection versus lobectomy for stage I non-small cell lung cancer: an appropriate choice in elderly patients?. Surg Today.

[9] Tsutani Y, Miyata Y, Nakayama H, Okumura S, Adachi S, Yoshimura M, Okada M (2014). Appropriate sublobar resection choice for ground glass opacity-dominant clinical stage IA lung adenocarcinoma: wedge resection or segmentectomy. Chest.

[10] Gu Z, Wang H, Mao T, Ji C, Xiang Y, Zhu Y, Xu P, Fang W (2018). Pulmonary function changes after different extent of pulmonary resection under video-assisted thoracic surgery. J Thorac Dis.

[11] Mori S, Shibazaki T, Noda Y, Kato D, Nakada T, Asano H, Matsudaira H, Ohtsuka T (2019). Recovery of pulmonary function after lung wedge resection. J Thorac Dis.

[12] Zhang Q, Wang Z, Jiang Y, Li F, Zhang Z, Cai H (2021). The application value of computed tomography in combination with intraoperative noninvasive percutaneous ultrasonic localisation of subpleural pulmonary nodules/ground-glass opacity in uniportal video-assisted thoracoscopy. Wideochir Inne Tech Maloinwazyjne.

[13] Kato H, Oizumi H, Suzuki J, Hamada A, Watarai H, Nakahashi K, Sadahiro M (2017). Thoracoscopic wedge resection and segmentectomy for small-sized pulmonary nodules. J Vis Surg.

[14] Zhang C, Lin H, Fu R, Zhang T, Nie Q, Dong S, Yang XN, Wu YL, Zhong WZ (2019). Application of indocyanine green fluorescence for precision sublobar resection. Thorac Cancer.

[15] Hoeijmakers F, Hartemink KJ, Verhagen AF, Steup WH, Marra E, Roell WFB, Heineman DJ, Schreurs WH, Tollenaar R, Wouters M (2021). Variation in incidence, prevention and treatment of persistent air leak after lung cancer surgery. Eur J Cardiothorac Surg.

[16] Miyasaka Y, Oh S, Takahashi N, Takamochi K, Suzuki K (2011). Postoperative complications and respiratory function following segmentectomy of the lung - comparison of the methods of making an inter-segmental plane. Interact Cardiovasc Thorac Surg.

[17] Chinese guidelines for diagnosis and treatment of primary lung cancer 2018 (English version). Chin J Cancer Res. 2019;31(1):1–28. 10.21147/j.issn.1000-9604.2019.01.01 PMC6433582 30996564

[18] Suzuki K, Watanabe SI, Wakabayashi M, Saji H, Aokage K, Moriya Y, Yoshino I, Tsuboi M, Nakamura S, Nakamura K (2022). A single-arm study of sublobar resection for ground-glass opacity dominant peripheral lung cancer. J Thorac Cardiovasc Surg.

[19] Mei J, Guo C, Xia L, Liao H, Pu Q, Ma L, Liu C, Zhu Y, Lin F, Yang Z (2019). Long-term survival outcomes of video-assisted thoracic surgery lobectomy for stage I–II non-small cell lung cancer are more favorable than thoracotomy: a propensity score-matched analysis from a high-volume center in China. Transl Lung Cancer Res.

[20] Endoh H, Tanaka S, Yajima T, Ito T, Tajima K, Mogi A, Shitara Y, Kuwano H (2010). Pulmonary function after pulmonary resection by posterior thoracotomy, anterior thoracotomy or video-assisted surgery. Eur J Cardiothorac Surg.

[21] Oparka J, Yan TD, Ryan E, Dunning J (2013). Does video-assisted thoracic surgery provide a safe alternative to conventional techniques in patients with limited pulmonary function who are otherwise suitable for lung resection?. Interact Cardiovasc Thorac Surg.

[22] Asakura K, Izumi Y, Kohno M, Ohtsuka T, Okui M, Hashimoto K, Nakayama T, Nomori H (2011). Effect of cutting technique at the intersegmental plane during segmentectomy on expansion of the preserved segment: comparison between staplers and scissors in ex vivo pig lung. Eur J Cardiothorac Surg.

[23] Charloux A, Quoix E (2017). Lung segmentectomy: does it offer a real functional benefit over lobectomy?. Eur Respir Rev.

[24] Fernandez-Rodriguez L, Torres I, Romera D, Galera R, Casitas R, Martinez-Ceron E, Diaz-Agero P, Utrilla C, Garcia-Rio F (2018). Prediction of postoperative lung function after major lung resection for lung cancer using volumetric computed tomography. J Thorac Cardiovasc Surg.

[25] Sugita Y, Kuroda H, Sakata S, Sakao Y, Okubo K (2021). How preserved regional pulmonary function after thoracoscopic segmentectomy in clinical stage I non-small cell lung cancers in right upper lobe. Gen Thorac Cardiovasc Surg.

[26] Chen L, Gu Z, Lin B, Wang W, Xu N, Liu Y, Ji C, Fang W (2021). Pulmonary function changes after thoracoscopic lobectomy versus intentional thoracoscopic segmentectomy for early-stage non-small cell lung cancer. Transl Lung Cancer Res.

[27] Arena R, Cahalin LP (2014). Evaluation of cardiorespiratory fitness and respiratory muscle function in the obese population. Prog Cardiovasc Dis.

[28] Liu W, Pan YL, Gao CX, Shang Z, Ning LJ, Liu X (2013). Breathing exercises improve post-operative pulmonary function and quality of life in patients with lung cancer: a meta-analysis. Exp Ther Med.

